# Supplementary material for: The role of Zambia’s expansive Inter-agency Coordinating Committee (ICC) in supporting evidence-based vaccine and health sector programming
Source: PLOS Glob Public Health. 2024 May 30;4(5):e0002820. doi: 10.1371/journal.pgph.0002820 (PMC11139285; doi:10.1371/journal.pgph.0002820)
Supplement: Attachment — Submitted filename: Zambia ICC Manuscript - Revisions Table - 02.29.2024.docx [file pgph.0002820.s002.docx]

**Edits & Revisions to Editor and Reviewers Comments**

*The role of Zambia’s expansive Inter-agency Coordinating Committee (ICC) in supporting evidence-based vaccine and health sector programming*

Sakas, et al.

| **Comment** | **Reviewer** | **Response** | **Line(s)** |
| --- | --- | --- | --- |
| Please include a complete copy of **PLOS’ questionnaire on inclusivity in global research** in your revised manuscript. Our policy for research in this area aims to improve transparency in the reporting of research performed outside of researchers’ own country or community. The policy applies to researchers who have travelled to a different country to conduct research, research with Indigenous populations or their lands, and research on cultural artefacts. The questionnaire can also be requested at the journal’s discretion for any other submissions, even if these conditions are not met. Please find more information on the policy and a link to download a blank copy of the questionnaire here: https://journals.plos.org/globalpublichealth/s/best-practices-in-research-reporting. Please upload a completed version of your questionnaire as Supporting Information when you resubmit your manuscript. | Editor | We have updated our submission to include our inclusivity in global research statement; please see attached. | N/A |
| Please amend your detailed Financial Disclosure statement. This is published with the article. It must therefore be completed in full sentences and contain the exact wording you wish to be published.  a. State the initials, alongside each funding source, of each author to receive each grant.  b. State what role the funders took in the study. If the funders had no role in your study, please state: “The funders had no role in study design, data collection and analysis, decision to publish, or preparation of the manuscript.”  c. If any authors received a salary from any of your funders, please state which authors and which funders.  If you did not receive any funding for this study, please simply state: “The authors received no specific funding for this work.” | Editor | See updated “Financial disclosures.”  *“This work was supported by the Bill & Melinda Gates Foundation, grant number OPP1195041 (awarded to MCF); salary support was provided to ZS, KR, KAH, RD, BD, ASE, KRI, WO, MCF, RAB, and a subaward was granted to WK. Pilot and proposal development funds were provided by Gates Ventures (MCF); salary support provided to KH, RAB, MCF. The Bill & Melinda Gates Foundation and Gates Ventures worked with the study team to provide technical assistance and assembled the Research Advisory Group. All other aspects—i.e., study design, materials, methodology, data collection and analysis, and manuscript writing—were done solely by the study team.”* | 502 |
| Please provide separate figure files in .tif or .eps format only and remove any figures embedded in your manuscript file. Please also ensure all files are under our size limit of 10MB.  For more information about figure files please see our guidelines:  https://journals.plos.org/globalpublichealth/s/figures  <https://journals.plos.org/globalpublichealth/s/figures#loc-file-requirement> | Editor | Noted - and fixed - for this submission. | N/A |
| Some material included in your submission may be copyrighted. According to PLOS’s copyright policy, authors who use figures or other material (e.g., graphics, clipart, maps) from another author or copyright holder must demonstrate or obtain permission to publish this material under the Creative Commons Attribution 4.0 International (CC BY 4.0) License used by PLOS journals. Please closely review the details of PLOS’s copyright requirements here: PLOS Licenses and Copyright. If you need to request permissions from a copyright holder, you may use PLOS's Copyright Content Permission form.  Please respond directly to this email or email the journal office and provide any known details concerning your material's license terms and permissions required for reuse, even if you have not yet obtained copyright permissions or are unsure of your material's copyright compatibility. | Editor | Thank you for prompting us to review the copyright information. Figures 1, 2, and 3 were created by Gates Ventures for use in this project, and in some form or another all appear on the exemplars.health platform. According to the [terms of use](https://www.exemplars.health/terms) on the Exemplars in Global Health website, “Exemplars grants you a limited, revocable license to copy and distribute the Content that appears on the Site only for non-commercial purposes, specifically, research, teaching and learning…”  To clarify the posted terms of use policy, we reached out to the Gates Ventures team, who confirmed our use is covered. We have included a signed permission form to include these figures. | N/A |
| Have the authors made all data underlying the findings in their manuscript fully available (please refer to the Data Availability Statement at the start of the manuscript PDF file)?  The PLOS Data policy requires authors to make all data underlying the findings described in their manuscript fully available without restriction, with rare exception. The data should be provided as part of the manuscript or its supporting information, or deposited to a public repository. For example, in addition to summary statistics, the data points behind means, medians and variance measures should be available. If there are restrictions on publicly sharing data—e.g. participant privacy or use of data from a third party—those must be specified.  Reviewer #1: No | Editor | Thank you for this reminder. While we have made focus group discussion guides, key informant interview guides, codebooks, and more publicly available via our [Open Science Framework (OSF) study page](https://osf.io/7ys4a/?view_only=739ca7a72f9749118b4aa3d2f7b655d9), the study team has made the decision to keep the underlying quotes available only on request. However, we have added additional quotes in a table that we hope will be of assistance.  According to the [PLOS data availability statement](https://journals.plos.org/plosone/s/data-availability), human research participant data may remain available upon request due to the sensitivity of the data. For this manuscript, we analyzed the interviews related to Zambia’s ICC – a small subset of individuals from an already small community of public health practitioners in early childhood immunization. Our concern in making the data publicly available is the ability for someone to recognize our participants. However, we are happy to work with researchers who have specific data requests; we have updated the data policy to be more specific.  In revised manuscript: *“The data are not publicly available as all data are confidential. The qualitative nature of this study—conducted among a small subset of key stakeholders in Zambia—renders the participants potentially identifiable. Upon request, the study team will work with requestors to provide specific, de-identified, materials arising from these interview transcripts.”* | 512 |
| **Reviewer 1:**  Although the authors specify that the primary case selection criterion was DTP1/3 coverage, it would help to further clarify why Zambia was chosen as the focus of this case study over the other two exemplars, especially since including one or both of the other countries could have enabled literal replication in the study design. | Reviewer 1 | We have added the below text and hope it clears up our selection:  *“Zambia was chosen for this specific case-study because the ICC, and the policy environment in general, emerged as a more significant theme in the qualitative data as compared to the other exemplar countries. While each country has a working ICC, key informants in Zambia spoke about the ICC and ZITAG frequently when asked about success factors that contributed to vaccination coverage. The emergence of the ICC as a unique factor prompted this study as to allow for a more thorough analysis of this topic.”* | 77-79 |
| Please consider including the key informant interview protocol and codebook as appendices. | Reviewer 1 | Thank you. We discussed your suggestion, but ultimately decided to omit this from the appendices because the documents are lengthy. All the requested documents are in our publicly available OSF study page that is cited in the body of the manuscript. They can be viewed and downloaded directly from that website. | N/A |
| In Lines 129-30, the authors mention that they used "constructs from existing implementation science frameworks" to develop their codebook. Please specify which frameworks were used and include citations. | Reviewer 1 | Thank you for directing us to this omission; we have added our frameworks, CFIR and CICI, along with their references, to the Methods section.  *“ We developed a codebook using a deductive approach, applying constructs from existing implementation science frameworks—notably the Consolidated Framework for Implementation Research (CFIR) and the Context and Implementation of Complex Interventions (CICI) frameworks [16, 17].”* | 139-142 |
| How did the research team ensure that interview transcripts were coded consistently? Were they double-coded? | Reviewer 1 | These are excellent points, and we have added text to address them:  *“To ensure consistency between coders, qualitative analysts performed regular intercoder reliability tests and attended frequent coding meetings to address discrepancies, discuss emerging themes, and add empirical codes to the codebook.”* | 144-146 |
| Line 134 mentions a review of the literature and historical documents - was this process different from the Gavi document review described immediately after? If so, please provide details of the search strategy and inclusion/exclusion criteria used to carry out these activities. | Reviewer 1 | As this was a different document review, we have added text to reflect this:  *“We used keywords (e.g., “Zambian ICC”, “ZITAG”, “Expanded Programme on Immunization”, “vaccination in Zambia”) to search Zambian government websites and academic journals.”* | 149-151 |
| Please consider including a reflexivity statement to orient readers to how the authors' positionalities have shaped the study design, data collection, and analysis activities. | Reviewer 1 | We appreciate the suggestion and agree with the reasoning behind this addition. However, we would like to gently push back on adding a reflexivity statement at this time. According to recent literature*, there is reason to suggest that inclusivity statements are not always an added benefit. We have just included the questionnaire on inclusivity in global health with this latest submission, and believe this gets at the information you are looking for. With this research and the questionnaire in mind, we, respectfully, have not included such a statement.   *Savolainen, J., Casey, P. J., McBrayer, J. P., & Schwerdtle, P. N. (2023). Positionality and Its Problems: Questioning the Value of Reflexivity Statements in Research. Perspectives on Psychological Science, 18(6), 1331-1338. <https://doi.org/10.1177/17456916221144988> | N/A |
| The authors might wish to include a table with more quotes from the key informant interviews, as this context could further support the reported findings. | Reviewer 1 | Thank you for this suggestion; we have added a table with pertinent quotes. | 233 |
| If possible to include, it would be interesting to read about how donors and external partners (other than Gavi) informed priority-setting, funding, and other ICC/ZITAG activities and decision-making processes. | Reviewer 1 | As the focus of this analysis was on operational mechanisms of the ICC, and not specific decision-making outcomes, we do not have information on those queries. The focus of our data collection was not on those specific outcomes, and we are unable to comment on that topic specifically. Any information we have related to other donors and external partners – and any other process – can be found in the main case study, which is cited in the manuscript.  In our conclusion, we acknowledge that more research is needed to explore these important questions: *“More research is needed to explore the role of external donors within ICC decision-making, the differences between ICCs from different countries, the costs related to ICC activities, and the integral relationship between ICCs and NITAGs.”* | 463-465 |
| Did the data reveal anything about the ICC/ZITAG's roles in implementing vaccination campaigns? | Reviewer 1 | We reviewed our data, and ultimately made no changes as there was not enough data on this specific query. Although we find that vaccination campaigns were an important part of Zambia’s vaccine programming strategy, we unfortunately do not have more information regarding the ICC’s role in implementing the campaigns. We have other papers that speak more to the immunization campaigns, including our main case study on Zambia & a paper on the demand environment across the 3 exemplar countries.  **Citations:**  Micek K, Hester KA, Chanda C, Darwar R, Dounebaine B, Ellis AS, et al. Critical success factors for routine immunization performance: A case study of zambia 2000 to 2018. Vaccine: X. 2022; 11:100166. doi:10.1016/j.jvacx.2022.100166  Hester, K., Sakas, Z., Ogutu, E.A., Dixit, S., Ellis, A.S., Yang, C., Chanda, C., Freeman, M.C., Orenstein, W.A., Sarr, M., Bednarczyk., R.A. (2023). Critical interventions for demand generation in Zambia, Nepal, and Senegal with regards to the 5C psychological antecedents of vaccination. Vaccine X. [Access: https://doi.org/10.1016/j.jvacx.2023.100341] | N/A |
| Please elaborate on the ICC/ZITAG's roles in shaping vaccine equity, as this was noted in Gavi's 2018 joint appraisal but not really reflected in the reported findings. | Reviewer 1 | Unfortunately, we don’t have qualitative data on the ICC & equity specifically, as we asked questions related to operational rather than programmatic outcomes. We have added more on equity to the discussion and limitations sections to compensate for this.  Additionally, as seen in Fig. 3, these improved operational mechanisms did lead to improvements in regional-level coverage and therefore equity.  We added more starting with this sentence: *“Although we did not have data that spoke specifically to the impact of the ICC on equity, our Zambia case study – which includes all key success factors including the ICC - shows improvements in vaccination coverage across all regions and in both rural and urban areas [11].”*  Other text added starts with: *“Additionally, because we only conducted one round of qualitative data collection, we did not have a chance to collect additional information after our analysis to gain more insight on specific topics that emerged during analysis (e.g., equity, vaccination campaigns, external partnerships).”* | 419-422  &  451-454 |
| I strongly suggest drafting a formal discussion section (separate from the findings and conclusion) to further elaborate on the transferable lessons and limitations of this study. Potential questions to explore in this section include: | Reviewer 1 | We have taken this suggestion and added a discussion section, which starts on page 22. Additionally, we moved some information from the results section to this new discussion section. | 383 |
| - The "so what" - what should the readers of this paper take away from this case study? The transferable lessons are a good start, but more detail is needed regarding who (or what institutions) should act on these lessons. Although data collection concluded prior to the COVID-19 pandemic, it may also be worth reflecting on how the roles and capacities of ICCs/NITAGs should evolve in response to the pandemic or other large-scale health emergencies. | Reviewer 1 | We ultimately do not have the ability to answer some aspects of this comment due to the mandate and the scope of the Exemplars in Health project as “hypothesis generating”. However, we have tried to address this comment as far as our data allows, and we’ve added more related to this in the discussion section, which starts on page 22. | See new Discussion on Page 22 |
| Add to Discussion-  Related research: The authors should consider moving their findings on ICCs in other African countries here. | Reviewer 1 | Addressed by moving related paragraph to the new Discussion section. | 424-435 |
| Add to Discussion-  What surprised you about the Zambian context? Were there any unexpected findings? | Reviewer 1 | Thank you for this feedback. The main objective of this project was “hypothesis generating” and to gain knowledge about drivers of catalytic growth in under 1 childhood vaccine delivery. Given the broad and open-ended data collection that was undertaken the study team, we took a grounded approach to this study which didn’t leave much space for surprises, per say. Additionally, we purposefully performed literature and document reviews after a scoping visit to the country to ensure the core study team had no preconceptions about direction of the research prior to commencing this study. | N/A |
| Add to Discussion-  Are there any questions your study did not answer? What lines of inquiry should future studies pursue? | Reviewer 1 | Due to the “hypothesis generating” nature of this study, we ended up with several areas of interest that we hope to further pursue. The publication of these findings will support future research and as well as program implementation efforts.  In our conclusion, we acknowledge that more research is needed to explore these important questions: *“More research is needed to explore the role of external donors within ICC decision-making, the differences between ICCs from different countries, the costs related to ICC activities, and the integral relationship between ICCs and NITAGs.”* | 463-467 |
| **Reviewer 2:**  Page 17 Section 3.1.2. I suggest that the authors consider incorporating a more balanced discussion by including direct quotes and additional insights from key informants who raised concerns regarding the expansion. Their perspectives shed light on the various issues associated with the proposed expansion and would contribute to a comprehensive understanding of the stakeholders' viewpoints. | Reviewer 2 | Thank you for this feedback. We went back to our data and reviewed the pertinent transcripts in an attempt to incorporate your feedback. Ultimately, our review confirmed that the participants interviewed for this study did not raise concerns regarding the expansion. Our topic guides did not specifically ask about concerns, which may be a gap in our data.  We have added the following text to this paragraph to address your concern (added the text in **bold**):  *“For example, key informants interviewed for this study spoke positively about the expanded mandate and cross-sectoral approach,* ***and did not mention any concerns during interviews,*** *a recent Gavi evaluation revealed that members of the ZITAG and EPI-TWG had some concerns over the recent changes.”* | 407-410 |
| Page 19 Section 3.2. Adjust the citation in Section 3.2 to adhere to the specified guidelines. Please ensure consistency and accuracy. | Reviewer 2 | Thank you for noticing this! We have addressed it. It should be Reference 7. | 544 |
| Page 22 Section 3.4. The authors could enhance the depth of Section 3.4, to include the practical application of the transferable lessons in various countries and contexts. Specific examples and contexts should be highlighted, elucidating the factors that contribute to the adaptability and relevance of these lessons in diverse settings. | Reviewer 2 | Thank you for this comment. We have added some text to address this.  Added text:  “*Countries looking to strengthen their policy environments, streamline decision making, improve communication between national-level stakeholders, or advocate for additional health funding may do so by adapting their ICCs based on the following transferable lessons…”*  The case study we published on Zambia has this information and includes some overarching information on the ICC within the country context.  **Case study citation:**  Micek K, Hester KA, Chanda C, Darwar R, Dounebaine B, Ellis AS, et al. Critical success factors for routine immunization performance: A case study of zambia 2000 to 2018. Vaccine: X. 2022; 11:100166. doi:10.1016/j.jvacx.2022.100166 | 393-395 |
| Page 23 Section 4. Section 4 should include an explanation of how the identified challenges may impact the generalizability of the findings. This discussion will provide clarity on the limitations and considerations necessary when extrapolating the results to broader contexts. | Reviewer 2 | We addressed this by adding a limitations paragraph:  *“These limitations may influence the generalizability of these findings. Further research is required to assess the potential impact of the transferable lessons described above. Similar analyses examining the ICCs of other countries with both low and high immunization coverage may improve the generalizability of this work.”* | 437-446 |
| Page 23 Section 4. Further clarification is needed in Section 4 to elaborate on challenges related to recall bias. The connection between these challenges and the limitations in accessing historical records should be explicitly explained, offering a more thorough understanding of potential biases in the data. | Reviewer 2 | Added text to address this comment:  *“Third, using qualitative methods to understand prior historical events was challenging, as interviewees often spoke about current experiences rather than discussing historical factors. Additionally, asking about historical events likely introduced some recall bias due to the long study period; participants may not have clearly remembered the inquired events and experiences.”* | 441-445 |
| Page 24 Section 5. The authors should reiterate and provide a clear link between the research objectives and the methods employed. Furthermore, the authors should consider how the subsequent findings are analyzed in the context of these RQs, demonstrating how the study addresses its overarching research goals. | Reviewer 2 | Thank you for this feedback - we have added text to address this comment:  *“Through qualitative research methods, this study sought to further understand the factors that positively impacted Zambia’s vaccination coverage, including the importance of a strong policy environment to support decision-making and strategic planning.”* | 457-459 |
